# A novel assessment considering spatial and temporal variations of water quality to identify pollution sources in urban rivers

**DOI:** 10.1038/s41598-021-87671-4

**Published:** 2021-04-22

**Authors:** Sihang Yang, Manchun Liang, Zesheng Qin, Yiwu Qian, Mei Li, Yi Cao

**Affiliations:** 1 Institute of Public Safety Research, Department of Engineering Physics, Beijing Key Laboratory of City Integrated Emergency Response Science, Tsinghua University, Beijing, China; 2 Environmental Safety Business Division, Beijing GSafety Technology, Co., Ltd., Beijing, China; 3 Hefei Institute for Public Safety Research, Tsinghua University, Hefei, China

**Keywords:** Environmental monitoring, Environmental impact

## Abstract

It’s vital to explore critical indicators when identifying potential pollution sources of urban rivers. However, the variations of urban river water qualities following temporal and spatial disturbances were highly local-dependent, further complicating the understanding of pollution emission laws. In order to understand the successional trajectory of water qualities of urban rivers and the underlying mechanisms controlling these dynamics at local scale, we collected daily monitoring data for 17 physical and chemical parameters from seven on-line monitoring stations in Nanfeihe River, Anhui, China, during the year 2018. The water quality at tributaries were similar, while that at main river was much different. A seasonal ‘’turning-back” pattern was observed in the water quality, which changed significantly from spring to summer but finally changed back in winter. This result was possibly regulated by seasonally-changed dissolved oxygen and water temperature. Linear mixed models showed that the site 2, with the highest loads of pollution, contributed the highest (β = 0.316, *P* < 0.001) to the main river City Water Quality Index (CWQI) index, but site 5, the geographically nearest site to main river monitoring station, did not show significant effect. In contrast, site 5 but not site 2 contributed the highest (β = 0.379, *P* < 0.001) to the main river water quality. Therefore, CWQI index was a better index than water quality to identify potential pollution sources with heavy loads of pollutants, despite temporal and spatial disturbances at local scales. These results highlight the role of aeration in water quality controlling of urban rivers, and emphasized the necessity to select proper index to accurately trace the latent pollution sources.

## Introduction

Surface water polluted with toxic chemicals and rivers eutrophicated with excess nutrients are of great environmental concerns worldwide. Toxic chemicals and biologically available nutrients discharged in excess to rivers can cause loss of oxygen, toxic algal blooms, fish deaths, loss of aquatic plant beds and coral reefs, finally leading to biodiversity losses^1^. In addition, such nutrient enrichment would seriously threaten the safety of drinking water, as well as the water for industry, agriculture, and recreation and other purposes^2^. In conclusion, human activities severely affected river water quality and hydrological cycles^3^. This can consequently result in negative effects on the aquatic ecosystems, including accelerated degradation of river ecosystems and damaged ecological values of freshwater^4^.

Because river water quality is typically highly variable through time, the attribution of changes induced by human activities is difficult to measure, especially when continuous and rapid monitoring through all year round is not available^5^. Therefore, water quality parameters in river were auto-monitored in order to characterize these short-lived but environmentally-significant events^5–8^. The high frequency (i.e., daily or sub-hourly monitored) sensors were used of river-water quality parameters, such as pH, turbidity, temperature, electrical conductivity, dissolved oxygen and fluorescence^9^. In addition, the number of water quality parameters that can be accurately monitored in situ has also increased, including for instances, dissolved organic matter, dissolved organic carbon, nitrate and chlorophyll-a^7,10^. Other technological advances, such as colorimetry on river banks for determinants, also permit sampling and rapid chemical analysis at trace concentrations^11^. Consequently, greater opportunity arises now to obtain temporal and spatial chemograph shapes of river water quality without compromising by under-sampling, and thus could avoid artefacts related to sampling in model parameterizations^12^. The water monitoring stations were often used, but it would produce a large quantity of data sets too complex to interpret and often not fully explored.

The temporal-spatial changes in water quality are fundamental to evaluate variations of river water pollution, due to natural or anthropogenic inputs of pollutant sources. River water pollutants usually come from highly seasonal-dependent transport pathways, such as ditches and creeks discharges, groundwater seepage, storm water runoff and atmospheric deposition^2^. Previous studies found that water quality at Yuqiao Reservoir Basin in China was clustered temporally by hydrological conditions (i.e., river water flow), but spatially by water quality parameters (i.e., water temperature, pH, suspended solids, total hardness, dissolved oxygen, nitrate and total phosphorus)^13^. Driving effects of water quality and hydrological factors varied significantly across spatial distributions, due to differences in geographical environments, water salinity and water pollution^4^. Such changes in water quality, rather than hydrological factors, was documented to further affect the spatial variations in the food-web stability^4^. At Copenhagen, Denmark, high level of water consumption across 28 well fields caused a steady increase in sulfate and calcium in water^14^. Significant and positive spatial correlations among water quality physical and chemical parameters were observed across 48 monitoring stations for the aquatic ecosystem in the first-grade freshwater ecoregions of Ji’nan, China^4^.

Hefei was the first pilot city for water ecological civilization in China, as well as the study city to build “healthy water ecological communities”, aiming to promote city sustainable development. It should absolutely benefit people’s life in China to successfully restore the ecology in healthy water ecological communities. Nanfeihe River was the Mother River of Hefei, which eventually flowed into the severely eutrophicated Chaohu Lake. Nutrients, heavy metals, hydrocarbons and pesticides were common contaminants of the NanfeiheRiver water pollution, coming from both point and non-point sources. These contaminants to the river were mainly from nearby ditches and creeks, aquatic weed control, storm water runoff, natural organic inputs and atmospheric deposition. The simultaneous degradation of river water quality has caused significantly changed species composition, resulting in overall deteriorated health status of aquatic communities within this region. Great efforts, including the development of the river water on-line monitoring stations, have been devoted to protecting the health of the Nanfeihe River and preventing its repeated pollution during the past two decades.

In this study, we collected daily monitoring data for 17 physical and chemical parameters from seven on-line monitoring stations in Nanfeihe River, Anhui, China, during the year 2018. We focused on the water qualities of urban rivers but not the whole aquatic ecosystems, which simplified the evaluation of pollution effects. We targeted to examine overall trends across many catchment fields and simultaneously identify the potential pollution sources of the main river. We also compared two indexes, i.e., City Water Quality Index (CWQI) and water quality, to select a better one to reflect the effect of heavy pollution loads from tributaries on the main river. The specific objectives in this study were to:

- Evaluate temporal and spatial patterns in water quality across seven on-line monitoring stations.
- Identify which parameters controlled the changes of water quality in the studied river fields and determine permit compliance for water pollutants.
- Explore critical indicators to identify pollution sources and quantify the level of anthropogenic activities’ influences on the river water qualities.

To address these objectives, we explored the hypothesis that emissions of water pollutants would mainly determine the temporal and spatial patterns in river water quality. We also hypothesized that the water pollutant with the highest amount would be the critical indicator when identifying potential pollution sources at the main river. Overall, our work would provide a simple and reliable approach to evaluate potential pollution sources of Nanfeihe River of Anhui Province in China and may be useful for the pollution prevention and control, despite temporal and spatial disturbances.

## Materials and methods

### Study site

Nanfeihe River was located in Hefei, Anhui province (31°30′–32°37′N and 116°40′– 117°52′E) in Central China, encompassing a drainage area of 1544 km^2^. This river originated from the southern part of Great Qian mountain and eventually flowed into Chaohu Lake, with Yangtze-Huaihe River Basin hilly areas spanning the whole region. This city experienced typically subtropical monsoon climate. The average annual temperature was 15.7 °C, with the lowest average monthly temperature of 2–3 °C in January and the highest average monthly temperature of 28–30 °C in July. The average annual precipitation was 964.4 mm, mostly raining from May to September. Hefei City was a typical developing city in China, with an area of 11,445 km^2^ and a population of 7.4 M. Drinking water safety, human health and well-being in Hefei, especially in Nanfeihe River Basin, were increasingly threatened, mainly due to rapid industrial development and urbanization and its tightly-related water pollution.

In this study, we selected a well-developed portion of the Nanfeihe River Basin for study, including four tributaries of Sili River (Site 1), Banqiao River (Site 2), ErshiliBu River (Site 3 & 4), Dianbu River (Site 5), and main stream of Nanfeihe River (Site 6 & 7) (Fig. S1). The upstream catchment of Nanfeihe River was a mix of unban residential land and industrial land, while the downstream was a traditional farming land. For this reason, the water quality of Nanfeihe River directly affected irrigation water safety in Hefei. Seven water quality on-line monitoring stations examined and recorded in situ water quality every day in the year 2018, covering the tributaries and main river of the Nanfeihe River (Fig. S1).

### Monitoring and sampling methods

Compared to existing laboratory-based methods, online monitoring system is fast to develop operational response and can provide in real time public health protection^15^. They were commonly used to monitor water quality parameters, including temperature, pH, turbidity, flow and chlorine, etc.^16^. On-line monitoring stations had its advantages in process control of water pollution, regulatory compliance and early warning systems for detecting water pollutants^17^. One of the most important goal of this method was to detect, in sufficient time, the high-impact but low-probability pollution events, in order to safeguard the public. The trending for developing the on-line monitoring stations was to detect deliberate and accidental pollution events, with few false negatives and positives^18^. The on-line monitoring stations were also required to be inexpensive and easily maintainable, as well as being easily integrated into network operations^18^.

In this study, daily-monitored data sets over the year 2018, from seven water quality on-line monitoring stations comprising 17 water quality parameters, were selected for analysis: chemical oxygen demand (COD), permanganate index (COD_Mn_), oil pollutant concentration, ammonia–nitrogen (NH_4_^+^-N), total nitrogen (TN), total phosphorus (TP), dissolved oxygen (DO), pH, Cd concentration, As concentration, Pd concentration, Cr concentration, temperature, flow velocity, flow rate, conductivity and turbidity.

Flow velocity and flow rate were routinely monitored, using a radio flow meter (Stalker II SVR V1.0) and traditional flow meter (LS25-1). Water turbidity, conductivity, DO, temperature and pH was measured by conventional five parameter water quality analyzer (HACH, sc-1000). Concentrations of NH_4_^+^-N, TN and TP were measured by a spectrophotometer (DR5000). COD, COD_Mn_ and oil pollutants were measured by a spectrolyzer (HR UV–VIS). We used an atomic absorption spectrophotometer (Thermo M6) to measure concentrations of heavy metals, including Pb, Cr, Cd and As. We integrated all these analyzers in an online water quality monitoring station in situ. In these monitoring stations, 50 ml water samples were firstly pumped by a self-priming pump equipped with float bowl, and then transported to a sample cup, from which the water samples were transported to each analyzer and detected at the same time. During the interval of continuously-detected samples, all the pumps, water inlet pipes and sensors were automatically washed by ionic free water. All the data were transported automatically via the internet to a server terminator at Hefei. Water temperature data at site 1 were missing, as well as the water conductivity at site 7.

### Water quality standards and data analysis

Environmental quality standards for surface water of China (No. GB3838-2002) stipulated that class III water quality standard for surface water, including river water, should have 6 < pH < 9, DO ≥ 5 mg/L, COD_Mn_ ≤ 6 mg/L, COD ≤ 20 mg/L, NH_4_^+^-N ≤ 1.0 mg/L, TP ≤ 0.2 mg/L, TN ≤ 1.0 mg/L, oil pollutants ≤ 0.05 mg/L, As ≤ 0.05 mg/L, Pb ≤ 0.05 mg/L, Cr ≤ 0.05 mg/L and Cd ≤ 0.005 mg/L. We used these standards to calculate City Water Quality Index (CWQI), of which the lower the value, the better the city water quality.

We first calculated CWQI(xi) for each parameter:

1 $$\mathrm{CWQI}(x_{i})=\frac{{c}_{monitor}({x}_{i})}{c_{III}({x}_{i})}$$

${c}_{monitor}$ represented the on-line monitored values of each parameter and ${c}_{III}$ represented the class III water quality standard for each parameter. This equation was applied to calculate CWQI index of COD, COD_Mn_, oil pullutants, NH_4_^+^-N, TN, TP, As, Cd, Cr, Pb.

For DO, the CWQI(DO) was calculated as:

2 $$\mathrm{CWQI}(\text{DO})=\frac{{c}_{III}(DO)}{{c}_{monitor}(DO)}$$

${c}_{III}(DO)$ represented the class III water quality standard for DO and ${c}_{monitor}(DO)$ represented the on-line monitored values of DO.

For pH, the CWQI (pH) was calculated as:

3 $$\mathrm{CWQI}(\mathrm{pH})=\frac{7.0-{pH}_{monitor}}{7.0-{pH}_{low}}, \quad \mathrm{ when} \; \; \text{pH} < 7$$

4 $$\mathrm{CWQI}(\text{pH})=\frac{{pH}_{monitor}-7.0}{{pH}_{high}-7.0}, \quad \mathrm{ when} \; \; \text{pH} \ge 7$$

${pH}_{low}$ referred to the lower limit of class III water quality standard, ${pH}_{high}$ referred to the higher limit of class III water quality standard.

CWQI of a certain river was calculated as:

5 $$\mathrm{CWQI}=\sum_{i=1}^{n}CWQI({x}_{i})$$

$CWQI({x}_{i})$ represented CWQI values of each water quality parameters on-line monitored.

We conducted the *Pearson* correlations between each pair of the water quality parameters collected at each monitoring site. Values were considered significant at *P* < 0.050. Principal Component Analysis (PCA) was used to measure the relative importance of each water quality parameter. Non-metric multidimensional scaling (NMDS) was performed to show the temporal variations of water quality. Forbenius Euclidean distance was calculated to quantify the differences of the water quality among different monitoring stations. On-way analyses of variance testing (ANOVA) were conducted to measure the effects of seasons on water quality parameters. All the analysis was performed using functions in the vegan (v. 2.5.5) packages in R (v. 3.6.1) (http://www.r-project.org).

The linear mixed model (LMM) analysis was conducted to establish linkages between water quality parameters and the water quality at tributaries, as well as their contributions to the CWQI index and the water quality at main river. In this LMM analysis, the water quality parameters and the water quality were used as predictors to examine their effects on CWQI and water qualities at main river. Standardized coefficient and *P* value for each path was reported from each LMM. Positive standardized coefficients suggested that the predictors have positive effects on CWQI or water quality, and negative standardized coefficients suggested negative effects. The standardized coefficient values indicated the relative importance of water quality at Site 1 to Site 6 on CWQI or water quality at main river. If the absolute value of the standardized coefficient was high, the specific corresponding site could be highly possible the potential pollution source. When *P* values were lower than 0.05, it suggested that the effects of certain predictors were significant. The effects of water quality parameters were significant on the water quality or CWQI index when *P* < 0.05. LMM was performed using functions in the pdist packages in R (V. 3.6.1).

Traditional water balance methods were also used to calculate the contributions of water quality at each site to that at main river. This method firstly calculated the total amount of pollutant emissions at each site, and then calculated the proportion of emissions at each site at tributaries to that at the main river.

## Results

### Spatial–temporal variations of water quality based on continuous on-line monitoring data

River quality was monitored for 297–319 days at the continuous on-line monitoring stations at both tributaries and main river. In terms of the comprehensive pollution of the seven monitoring stations, site 1 was the least polluted (CWQI = 497.3), and site 5 was the most polluted (CWQI = 1255.3), with site 2 (CWQI = 582.2), site 4 (CWQI = 655.9), site 7 (CWQI = 1059.6), site 3 (CWQI = 1172.2) and site 6 (CWQI = 1185.3) ranking from 2 to 6 among the cleanest sites.

**Figure 1:**
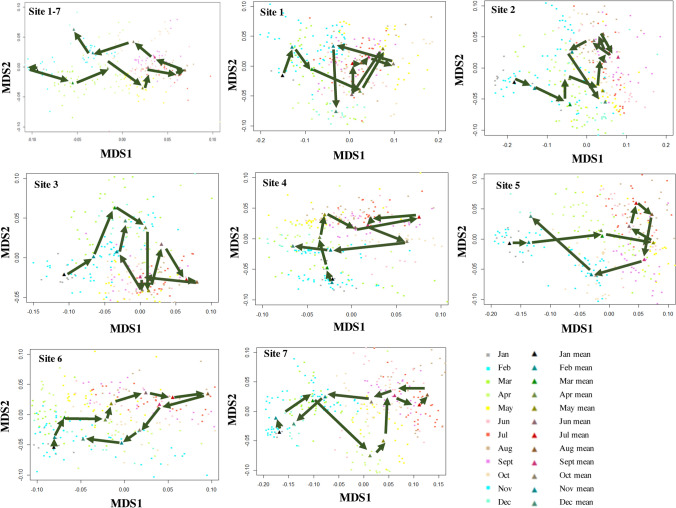
Non-Metric Multidimensional Scaling (NMDS) of samples from seven on-line monitoring stations. Samples from January to December were indicated by solid circles in different colors, with the solid triangles representing the mean values of different month.

**Figure 2:**
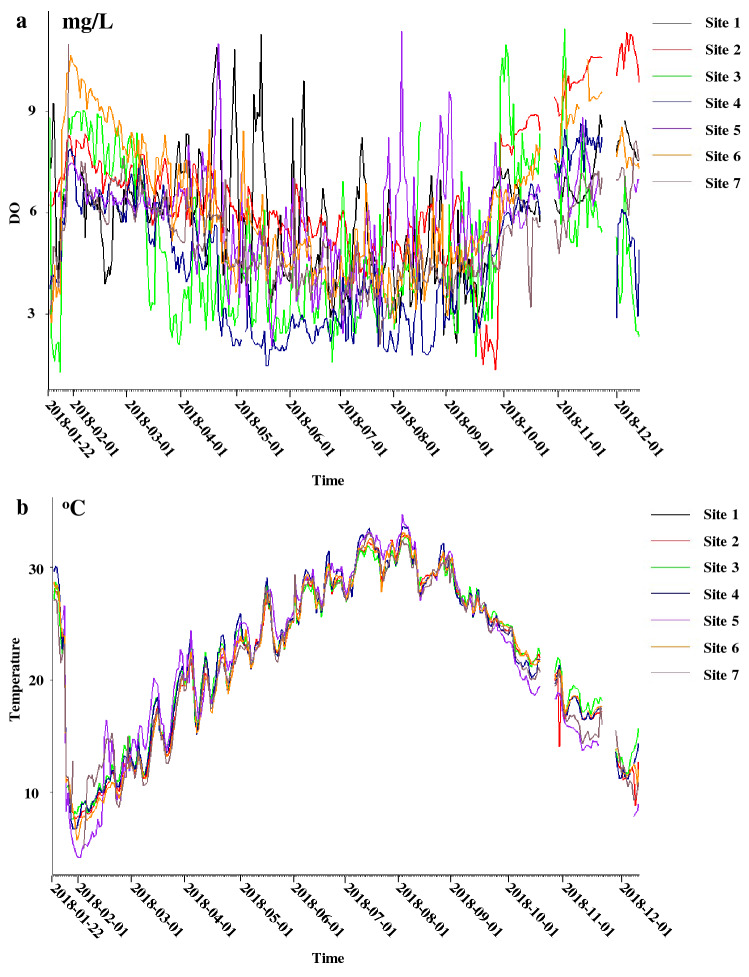
Time series of (**a**) DO and (**b**) water temperature at seven on-line monitoring stations during one-year period.

Most of those parameters were significantly different across four seasons and seven sites, at seven monitoring stations (Table S1, Figs. S2–S5). NMDS results revealed that the water quality varied seasonally and exhibited a “turning-back” pattern: the water quality changed significantly from spring to summer but finally changed back in winter (Fig. 1). This result paralleled the temporal variation pattern of DO and water temperature at all the on-line monitoring stations, where the concentrations of DO were low in summer (DO_summer_ = 2.94–5.37 mg/L) but high in winter (DO_winter_ = 6.10–8.79 mg/L) (Fig. 2a), and the water temperature was high in summer (T_summer_ = 29.08–30.25 °C) but low in winter (T_winter_ = 8.74–11.34 °C) (Fig. 2b).

We then examined how other water quality parameters varied spatially. Using Forbenius Euclidian matrix, we found that the distances were smaller and similar among site 1–6 at tributaries, but much larger when calculated between each site at tributaries and site 7 at main river (Table 1). This suggested that the similarities among each pair of six monitoring stations at tributaries were at the same extent, while the monitoring station at main river were much more different from those mentioned above. According to the GB3838-2002 standards, water quality of Class I and Class II are regarded as clean status, Class III is moderate pollution status, and Class IV and V are high pollution status. In general, all the water qualities at seven sites were classified as Class V, the high pollution status. All the water qualities at seven sites were identified as Class V, the high pollution status. Specifically, site 1 and 2 were mainly polluted by residential pollutants, which were typically polluted by COD and heavy metals. Site 3, 4 and 6 were mainly polluted by industrial pollutants (Figs. S2–S5), that is, NH_4_^+^-N, TN and TP. Site 5 and 7 were mainly polluted by agricultural pollutants, including organic matter, nutrient pollution and oil pollutants.

**Table 1:** Forbenius Euclidian matrix indicating differences among seven on-line monitoring stations.

	Site 1	Site 2	Site 3	Site 4	Site 5	Site 6	Site 7
Site 1							
Site 2	18,133.63						
Site 3	19,900.43	21,765.10					
Site 4	18,246.13	20,766.51	21,465.09				
Site 5	18,331.01	20,130.35	17,064.28	19,224.32			
Site 6	16,956.33	19,253.87	16,059.22	19,095.57	14,277.57		
Site 7	42,094.93	42,403.82	42,978.28	41,233.87	39,553.12	42,291.11	

### Correlations among water quality parameters

**Figure 3:**
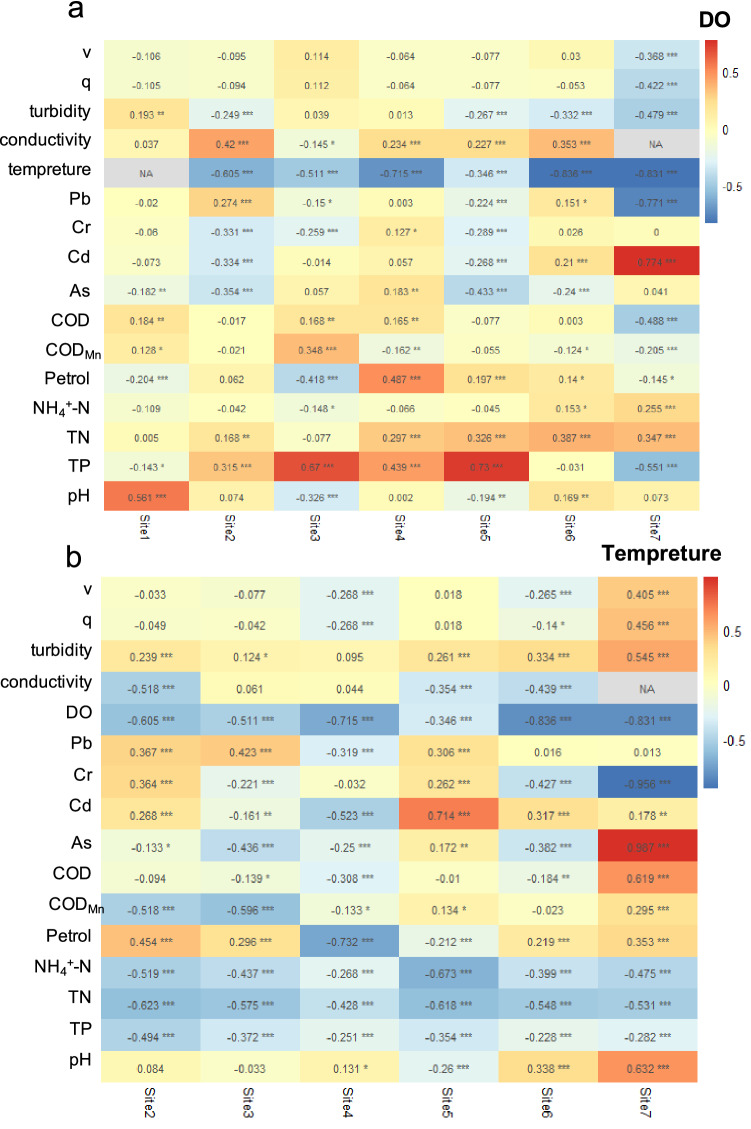
Heatmap for *Pearson* correlations between water quality parameters and (**a**) DO or (**b**) water temperature at seven on-line monitoring stations, respectively. Velocity, flow velocity; Q, flow; Turbidity, water turbidity; Conductivity, water conductivity; DO, dissolved oxygen; Temperature, water temperature; COD, chemical oxygen demand; COD_Mn_, chemical oxygen demand indicated by Permanganate Index; NH_4_^+^-N, ammonium; TN, total nitrogen; TP, total phosphorus; pH, water pH. Water temperature at site 1 and water conductivity at site 7 were missing.

We found that DO and water temperature were significantly correlated with water organic matters, heavy metals and nutrients (Fig. S6). *Pearson* correlations showed that DO concentration at site 1 was correlated positively with water pH (ρ = 0.561, *P* < 0.001), COD (ρ = 0.184, *P* = 0.002) and COD_Mn_ (ρ = 0.128, *P* = 0.034), but negatively with oil pollutants concentration (ρ = − 0.204, *P* < 0.001), As (ρ = − 0.182, *P* = 0.002) and TP (ρ = − 0.143, *P* = 0.018) (Fig. 3a). At site 2, DO significantly correlated with the concentration of heavy metal, i.e., Cr (ρ = − 0.331, *P* < 0.001), Pb (ρ = 0.274, *P* < 0.001) and Cd (ρ = − 0.334, *P* < 0.001), as well as TN (ρ = 0.168, *P* = 0.005), and TP (ρ = 0.315, *P* < 0.001) (Fig. 3a). At all the other five sites, DO was correlated with the concentration of both water nutrients and heavy metals. In addition, the temporal variation of temperature was also tightly and negatively coupled with that of water quality parameters at all sites (Fig. 3b), except that temperature data were missing at site 1.

### The contributions of latent pollution sources to the performance of CWQI and water quality at main river

**Figure 4:**
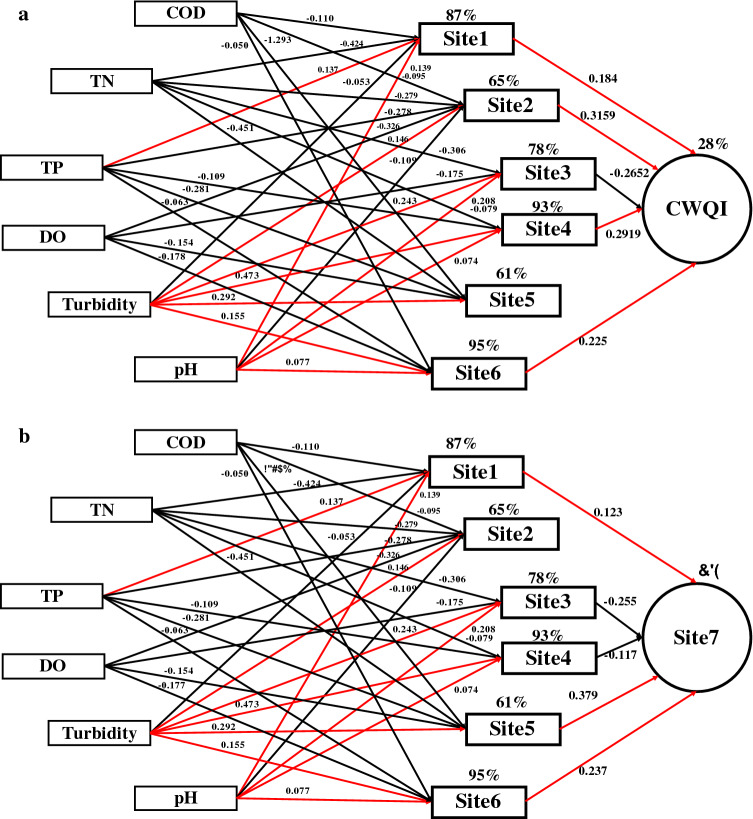
Linear mixed models (LMM) coupling selected water quality parameters and CWQI at main river. Regression R^2^ values from the permutation test were presented on the arrows, which were all significant (*P* < 0.050). Black arrows represented negative paths, and red arrows represented positive paths.

The linear mixed model analysis was conducted to assess the linkages between water quality parameters and the water quality at tributaries, as well as their contributions to the CWQI index and the water quality at main river. Using data from water quality parameters (i.e., COD, TN, TP, DO, turbidity and pH) and water quality, we generated a significant (*P* < 0.05) model (Fig. 4).

CWQI index at the main river was correlated with the water quality of site 2 (β = 0.316, *P* < 0.001) and site 4 (β = 0.292, *P* < 0.001) to similar extent, followed by site 3 (β = − 0.265, *P* = 0.003), site 6 (β = 0.225, *P* = 0.001) and site 1 (β = 0.184, *P* = 0.003) (Fig. 4a). Among those sites, site 2 observed the highest total amount of COD, Pb, Cr and Cd, while site 4 observed the highest total amount of COD_Mn_, NH_4_^+^-N, TN and As during 2018 (Figs. S2–S5). In addition, site 3 observed the highest total amount of TP and site 6 observed the highest total amount of oil pollutants. Those results indicated that CWQI index at the main river was mostly driven by the water quality at the most seriously polluted sites. Furthermore, not only the extent to which the parameters exceeded the standards, but also the number of parameters that exceeded the standards, was important when controlling the CWQI index. Surprisingly, the water quality of site 5, the nearest site to the main river monitoring station, was not significantly correlated with the CWQI index of the main river (β = − 0.139, *P* = 0.086). In contrast, the water quality of site 5 (β = 0.379, *P* < 0.001) showed the strongest effect on the water quality of main river, whereas the effect of site 2 was negligible (β = 0.103, *P* = 0.064) (Fig. 4b). This result suggested that the location of the pollution sources was an important factor on water quality. Generally, the water quality of sites 1–6 were significantly affected by COD, TN, TP, DO, turbidity and pH, explaining 28% of the CWQI index and 65% of the water quality at main river.

Traditional water balance methods were also used to examine the contribution of each site to the main river (Table S2). Water nutrients at main river, including NH_4_^+^-N, TN and TP, were mainly contributed by the nutrients from site 4 (12.6–14.9%), followed by site 1–3 and 6 (4.3–8.6%). The latent pollution sources of organic pollutants, represented by COD and COD_Mn_, and heavy metals, including As, Cd, Cr and Pb, were primarily site 2 and 4. Oil pollutants at main river was originated evenly from site 3–6 (7.2–8.7%).

## Discussion

### Effect-based urban surface water quality assessment to identify pollution sources

The water quality at severely polluted tributaries, but not the geographically nearest tributaries, contributed the most to CWQI index at main river (Fig. 4a). But the opposing result was observed when assessing the contributions to water quality at main river, where the geographically nearest water quality was the most important one (Fig. 4b). CWQI quantitatively measured the degree to which the urban surface waters were polluted and emphasized the ranking of water quality of different waters. Water quality mainly described the proportion of different components in certain surface waters. Therefore, when using CWQI index to identify latent pollution sources in urban rivers, the strategy would be to decrease the loads of heavily discharged pollutants at the most severely polluted sites, while the loads of pollutants at the nearest site would be mainly decreased when using water quality. The final purpose of the urban surface water quality assessment would be to accurately determine the site with the heavy loads and simultaneously formulate effective pollution reduction policies. So, from the perspective of policy making, using CWQI index as the output of LMM helped better with distinguishing the latent pollution sources. The importance of certain water quality parameter varied with seasons in contribution to water quality, therefore, seasonal variations should be given full thoughts when selecting parameters to establish pollutant load reduction goals and the developing total maximum daily loads^2^.

Indexes based on biological factors and physiochemical factors are two main types to assess polluted status in rivers and lakes^19,20^. CWQI in this study belonged to the latter type and predicted the risk of increased contaminant levels in urban rivers from 17 easy-to-measure parameters, which were related to the water pollution processes with varying levels of influence in multiples ways. The relationships between water quality parameters were also considered when using CWQI to generate better discrimination. In contrast, if considered in isolation, each parameter may not describe the polluted conditions accurately and comprehensively^20^. Climate, land surface characteristics, physiographic conditions, river morphology can also be important determinants of water quality, as well as pollutant source strength^20^. However, results from traditional water balance methods lacked the consideration of those aforementioned indicators in urban river systems, as well as the relationships among water quality parameters.

Water quality reflected influences of sewage inflow and spatial factors (e.g., landforms and land use) within the catchment^21,22^. Conductivity, pH and alkalinity were generally related with geological conditions^23^. The DO levels in river system were complexly dependent on water temperature, salinity, turbulence, depth, photosynthesis, aquatic plants respiration rates and breakdown rates of organic matter^24–27^. The predictability of water quality were reduced by the complexity among parameters, if each was considered in isolation^20^. A combined model considering the connections among water quality parameters, as well as hydrological and geographical conditions, would be the only way to overcome this problem^20^. Overall, water nutrients were mainly responsible for the growth of various macrophyte taxa^28^.

In present study, Site 2 & 4 experienced heavy pollution of organic matters and water nutrients, while site 5 observed much lower yearly average concentration of those pollutants. Our results suggested that industrial and residential lands were the major pollution sources for water quality deterioration, and the pollutions caused by agricultural activities were secondary in this Nanfeihe River basin. Consistent results were usually observed in highly-urbanized areas^29^. Similarly, the relatively weak association between agricultural activities and water contaminant concentrations were also found at eastern Massachusetts, USA^30^. In urban areas, continuously discharged city sewage with high concentration of pollutants played the primary role in deteriorating water quality, while vegetation coverage with low concentration of pollutants would improve the water quality^29^. Urban river systems in this study showed distinct water qualities from upstream to downstream, suggesting that the models we used were site specific. But the models developed here can be adapted for urban surface water systems elsewhere.

### Driving factors of spatial–temporal variations of water quality: hydrological factors, DO and water temperature

This study revealed the apparent pattern in the spatial variations of water qualities at seven monitoring stations, where those at tributaries were much similar than that at main river (Table 1). Pollution sources for site 1 & 2 were residential pollution, for 3, 4 & 6 were industrial pollution and for 5 & 7 were agricultural pollution. Despite different types of pollution, sites 1–6 located at tributaries observed similar distances among each other, while site 7 located at main river was much more different. Those differences could be explained by the hydrological factors, but not topography. The site 7 observed the highest velocity and the largest amount of flow, approximately tenfolds of those at tributaries. Consequently, the site 7 also observed the highest total amount of nutrients, organic matters and heavy metals, leading to significantly different water quality at main river compared to those at tributaries. In contrast, a previous study found that spatial trend of water quality was generally driven by non-point pollution sources, especially anthropogenic activities with respect to pollutant load and land use^29^. Such spatial differences could be attributed to different hydrological regimes and flow regulation, when large amount of flow would dilute the pollutants in river.

There was also a clear pattern in the temporal variations of water qualities, which significantly changed with DO. Specifically, the water qualities at seven monitoring sites in December were very similar to those in January, corresponding to high DO and low water temperature, whereas the water qualities in summer were very different, corresponding to low DO and high water temperature. It was a natural process that the solubility of oxygen was inversely related to temperature, since warmer water was more easily saturated with oxygen and thus holds less DO^31,32^. This could explain the temporal variation pattern, as more oxygen was available for reaction with the pollutants, especially heavy metals and organic pollutants, during winter periods^33^. Therefore, the characteristics of temporal variation in the water quality of Nanfeihe River Basin were possibly affected by DO and local climate characteristics in Central China: Spring comprised March to May, Summer comprised June to August, Autumn comprised September to November, and Winter comprised December to next February.

DO was strongly correlated with the organic matters, but not always highly correlated with total Kjeldahl-nitrogen in river water, and thus seasonal variations should be considered when using DO as an indicator to evaluate surface water quality^2^. In addition, pollution concentrations were negatively correlated with water temperature in urban rivers^29^. Temperature was significantly correlated with water quality parameters, such as turbidity and salinity, for the entire four seasons^34^. Two mechanisms could explain this phenomenon. First, microbes were facilitated to degrade water pollutants during warm seasons and therefore can effectively improve water quality^35^. Higher temperature in summer would thus lead to faster decomposition of organic matter, resulting in positive increase in correlations between temperature and organic matters^36^. Second, aquatic plants can uptake and temporarily store nutrients during warm growing seasons, thus affecting the cycling of nutrients in rivers^37^. In contrast, dilution effect during the wet summer seasons could lead to negative increase in correlations between temperature and mineral matters, such as alkalinity and salinity^38^. But water temperature was also documented to have very weak correlations with water quality parameters such as pH, color, DO, TOC and BOD^39^. Those inconsistencies could be partially caused by different river environments and partially caused by no seasonal analysis in some studies.

### Autocorrelation of water quality parameters

Water quality parameters were affected in a complex way with seasons and geographical locations^29^. Those studies, mainly focusing on the autocorrelation of water quality parameters in the water coastal aquifers, groundwater and urban aquatic environments, observed no autocorrelation in the water chemical parameters^40^, high spatial autocorrelation with sodium ions^41^, or most significant autocorrelation in bicarbonate^42^. Those results differed much from ours, since environmental parameters, especially geographical conditions, water pollutants and water salinity, could affect ionic content, resulting in variations in spatial distribution of water quality parameters across study areas.

## Conclusion

This study proposed a method to identify pollution sources, highlighting the importance of the effect-based urban surface water quality assessment. We first illustrated the temporal-spatial patterns of river water qualities of Nanfeihe River in Hefei, Anhui Province, China. River water qualities exhibited a “turning-back” temporal pattern from January to December, which was possibly affected by variations in water DO and temperature. River water qualities also exhibited a highly hydrologically-dependent spatial pattern, where the water qualities of main river were much different from those at tributaries. In addition, we found that site 2 with the largest loads of COD and heavy metals was the most important pollution source of the main river. This result indicated that CWQI index was a better index, than water quality, to reflect the pollution loads at the tributaries, offering good assessment skills for urban river systems polluted by anthropogenic sources. The ability to effectively identify pollution sources may become increasingly important, as potential industrial, residential and agricultural activities presented more challenges for water supply to comply with regulations and serve local communities. The effect-based assessment supplied critical information for policy makers to identify pollution sources in urban river systems caused by anthropogenic activities.

## Supplementary Information

Supplementary Information 1.

Supplementary Information 2.

## Data Availability

All the data were available in Supplementary Table 3.
