# Supplementary material for: A novel assessment considering spatial and temporal variations of water quality to identify pollution sources in urban rivers
Source: Sci Rep. 2021 Apr 22;11:8714. doi: 10.1038/s41598-021-87671-4 (PMC8062557; doi:10.1038/s41598-021-87671-4)

**A novel assessment considering spatial and temporal variations of water quality to identify pollution sources in urban rivers**

Sihang Yang^1,+^, Manchun Liang^1,+,*^, Zesheng Qin^2^, Yiwu Qian^3^, Mei Li^3^, Yi Cao^3^

^1^Institute of Public Safety Research, Department of Engineering Physics, Beijing Key Laboratory of City Integrated Emergency Response Science, Tsinghua University, Beijing, China

^2^Environmental Safety Business Division, Beijing GSafety Technology, Co., Ltd., Beijing, China

^3^Hefei Institute for Public Safety Research, Tsinghua University, Hefei, China

^+^These authors have contributed equally to this work.

^*^To whom correspondence may be addressed. E-mail: [lmc@tsinghua.edu.cn](mailto:lmc@tsinghua.edu.cn); Phone: 010-62795424; Fax: 010-62792863

**Running title:** On-line monitoring of river water quality

**Key words:** On-line monitoring station; Urban river; Water quality assessment; Pollution sources identification

**Type of paper:** Primary research article

**Table S1**. Summary of *P* values from one-way analyses of variance testing (ANOVA) for the effects of seasons on water quality parameters.

|  | **Site1** | **Site2** | **Site3** | **Site4** | **Site5** | **Site6** | **Site7** |
| --- | --- | --- | --- | --- | --- | --- | --- |
| **Velocity** | <0.001 | 0.127 | 0.001 | <0.001 | <0.001 | <0.001 | <0.001 |
| **Q** | <0.001 | 0.059 . | <0.001 | <0.001 | <0.001 | <0.001 | <0.001 |
| **Turbidity** | <0.001 | <0.001 | <0.001 | 0.005 | <0.001 | <0.001 | <0.001 |
| **Conductivity** | 0.257 | <0.001 | 0.021 | <0.001 | <0.001 | <0.001 | -- |
| **DO** | <0.001 | <0.001 | <0.001 | <0.001 | <0.001 | <0.001 | <0.001 |
| **Tempreture** | -- | <0.001 | <0.001 | <0.001 | <0.001 | <0.001 | <0.001 |
| **Pb** | <0.001 | <0.001 | <0.001 | <0.001 | <0.001 | 0.009 | <0.001 |
| **Cr** | <0.001 | 0.089 | 0.005 | <0.001 | <0.001 | <0.001 | <0.001 |
| **Cd** | <0.001 | 0.005 | <0.001 | <0.001 | <0.001 | <0.001 | <0.001 |
| **As** | <0.001 | 0.001 | <0.001 | <0.001 | <0.001 | <0.001 | <0.001 |
| **COD** | <0.001 | 0.009 | 0.394 | <0.001 | <0.001 | <0.001 | <0.001 |
| **COD_Mn_** | <0.001 | <0.001 | <0.001 | <0.001 | <0.001 | <0.001 | <0.001 |
| **Oil pollutants** | <0.001 | <0.001 | <0.001 | <0.001 | <0.001 | <0.001 | <0.001 |
| **NH_4_^+^-N** | <0.001 | <0.001 | <0.001 | <0.001 | <0.001 | <0.001 | <0.001 |
| **TN** | <0.001 | <0.001 | <0.001 | <0.001 | <0.001 | <0.001 | <0.001 |
| **TP** | <0.001 | <0.001 | <0.001 | <0.001 | <0.001 | <0.001 | <0.001 |
| **pH** | 0.027 | <0.001 | <0.001 | <0.001 | <0.001 | <0.001 | <0.001 |

Velocity, flow velocity; Q, flow; Turbidity, water turbidity; Conductivity, water conductivity; DO, dissolved oxygen; Temperature, water temperature; Pb, lead; Cr, chromium; Cd, cadmium; As, arsenic; COD, chemical oxygen demand; COD_Mn_, chemical oxygen demand indicated by Permanganate Index; NH_4_^+^-N, ammonium; TN, total nitrogen; TP, total phosphorus; pH, water pH.

**Table S2**. Contributions of each water pollutants from site 1 – 6 to site 7, calculated by traditional water balance methods.

|  | **Site1** | **Site2** | **Site3** | **Site4** | **Site5** | **Site6** | **Site7** |
| --- | --- | --- | --- | --- | --- | --- | --- |
| **Pb** | 10.5% | 15.3% | 6.8% | 12.4% | 8.4% | 6.8% | 39.8% |
| **Cr** | 12.7% | 17.1% | 1.6% | 17.5% | 9.1% | 7.3% | 34.7% |
| **Cd** | 5.6% | 11.2% | 2.7% | 9.1% | 6.1% | 3.3% | 62.0% |
| **As** | 5.8% | 7.7% | 5.0% | 10.3% | 4.4% | 4.3% | 62.6% |
| **COD** | 7.4% | 10.5% | 5.7% | 9.0% | 4.9% | 7.2% | 55.3% |
| **COD_Mn_** | 8.4% | 10.1% | 5.8% | 10.4% | 5.4% | 8.1% | 51.7% |
| **Oil pollutants** | 2.9% | 5.9% | 7.8% | 7.2% | 8.0% | 8.7% | 59.6% |
| **NH_4_^+^-N** | 7.8% | 6.2% | 4.3% | 14.3% | 2.7% | 7.3% | 57.4% |
| **TN** | 7.3% | 7.9% | 6.2% | 12.6% | 4.1% | 7.5% | 54.4% |
| **TP** | 7.1% | 7.8% | 7.3% | 14.9% | 3.1% | 8.6% | 51.2% |

Pb, lead; Cr, chromium; Cd, cadmium; As, arsenic; COD, chemical oxygen demand; COD_Mn_, chemical oxygen demand indicated by Permanganate Index; NH_4_^+^-N, ammonium; TN, total nitrogen; TP, total phosphorus; pH, water pH.

**Table S3.** Values of water pollutants’ concentrations, including COD, oil pollutants, Pb, Cr, Cd, As, DO, CODmn, NH_4_-N, TP, TN, flow velocity, flow rate, turbidity, Conductivity, pH and water temperature, which were measured in 2018 at Site 1-7.

This table is available as a supplementary dataset; Supplementary Table 3.xlsx.

**Figures**

**Figure S1**. Map of study area and water quality monitoring stations (Site 1-Site 7) in Nanfeihe River Basin. Different reaches of Nanfeihe river were marked with different colors according to the water quality classifications: red line represented worse than class V, orange line represented class V, yellow line represented class IV and green line represented better than class IV. This map was generated by the software *ArcGis for Personal Use* (ArcGis 10.5, URL: https://www.esri.com/en-us/arcgis/products/arcgis-for-personal-use/buy).


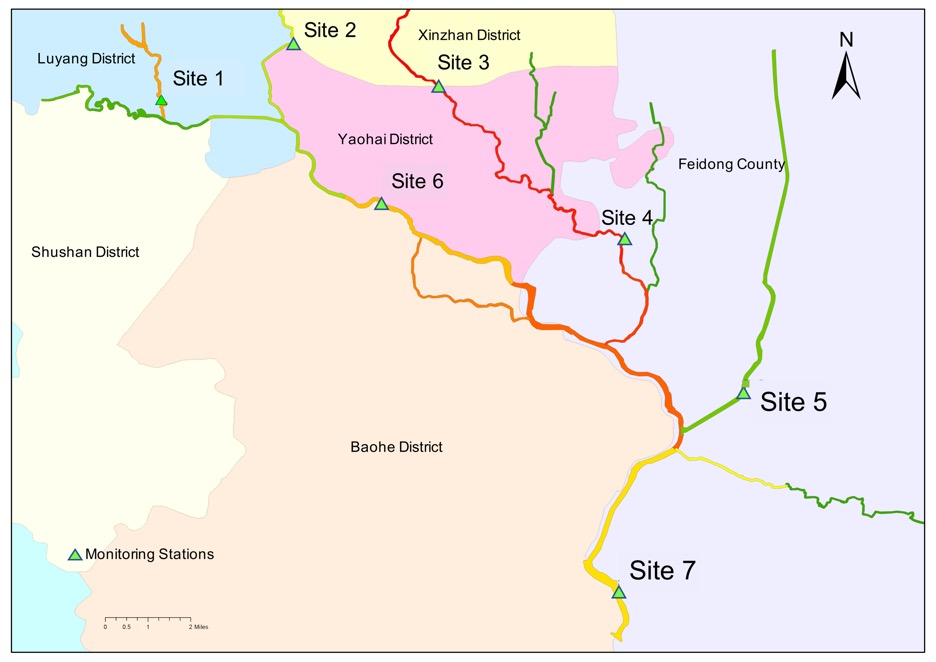


**Figure S2**. Boxplot for water physical quality parameters at seven on-line monitoring stations. Velocity, flow velocity; Q, flow; Turbidity, water turbidity; Conductivity, water conductivity.


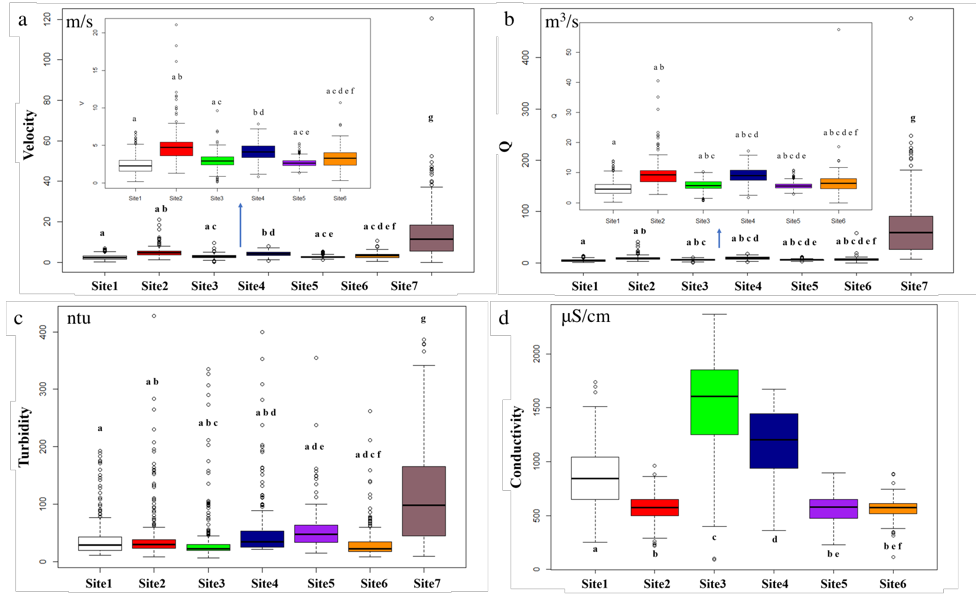


**Figure S3**. Boxplot for water heavy metal and As concentrations at seven on-line monitoring stations. Pb, lead; Cr, chromium; Cd, cadmium; As, arsenic.


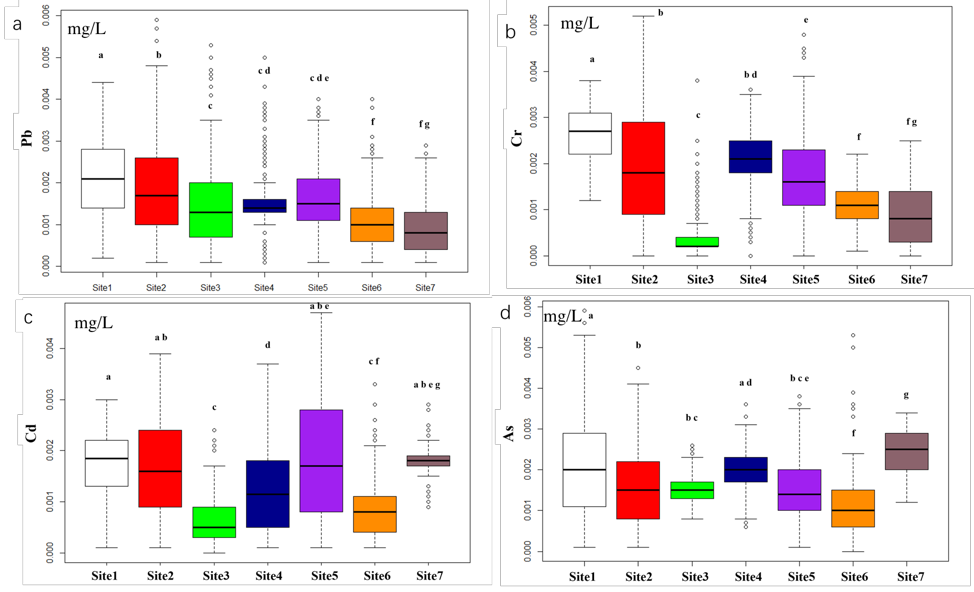


**Figure S4**. Boxplot for water organic pollutants and water pH at seven on-line monitoring stations. COD, chemical oxygen demand; COD_Mn_, chemical oxygen demand indicated by Permanganate Index; pH, water pH.


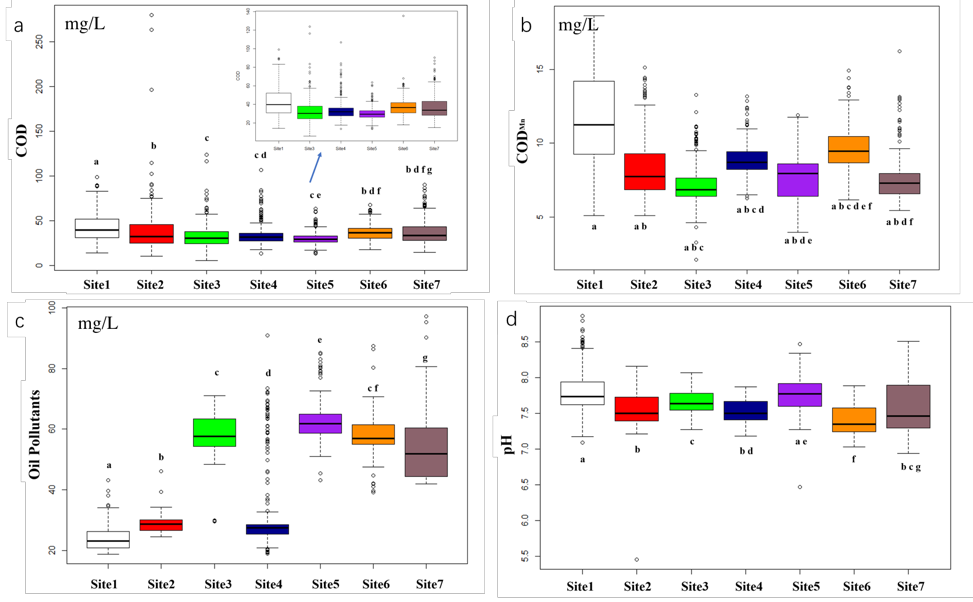


**Figure S5**. Boxplot for water nutrients at seven on-line monitoring stations. NH_4_^+^-N, ammonium; TN, total nitrogen; TP, total phosphorus; N/P ratio, nitrogen to phosphorus ratio.


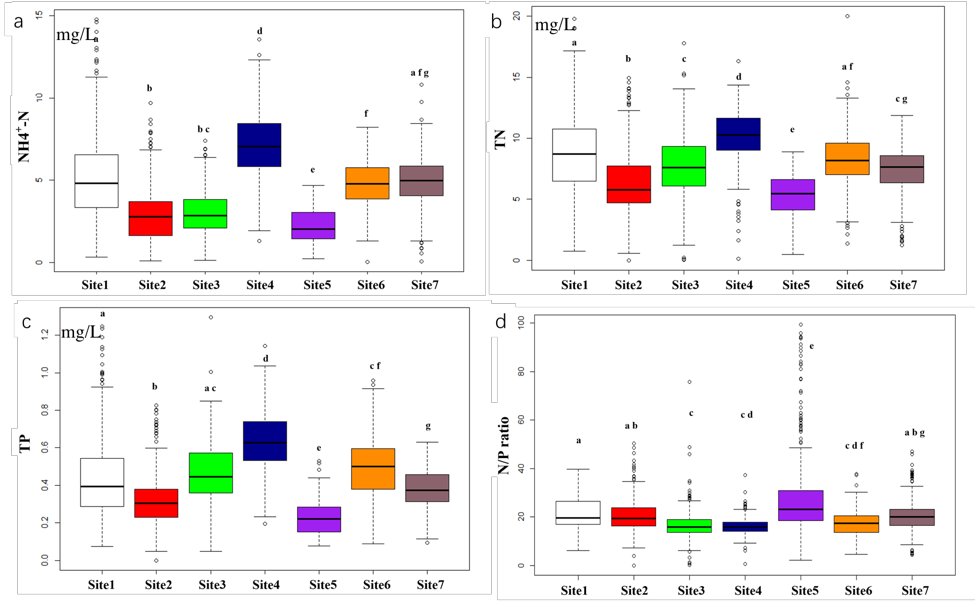


**Figure S6**. Principal Components Analysis (PCA) results for samples from seven on-line monitoring stations. Velocity, flow velocity; Q, flow; Turbidity, water turbidity; Conductivity, water conductivity; DO, dissolved oxygen; Temperature, water temperature; Pb, lead; Cr, chromium; Cd, cadmium; As, arsenic; COD, chemical oxygen demand; COD_Mn_, chemical oxygen demand indicated by Permanganate Index; NH_4_^+^-N, ammonium; TN, total nitrogen; TP, total phosphorus; pH, water pH.


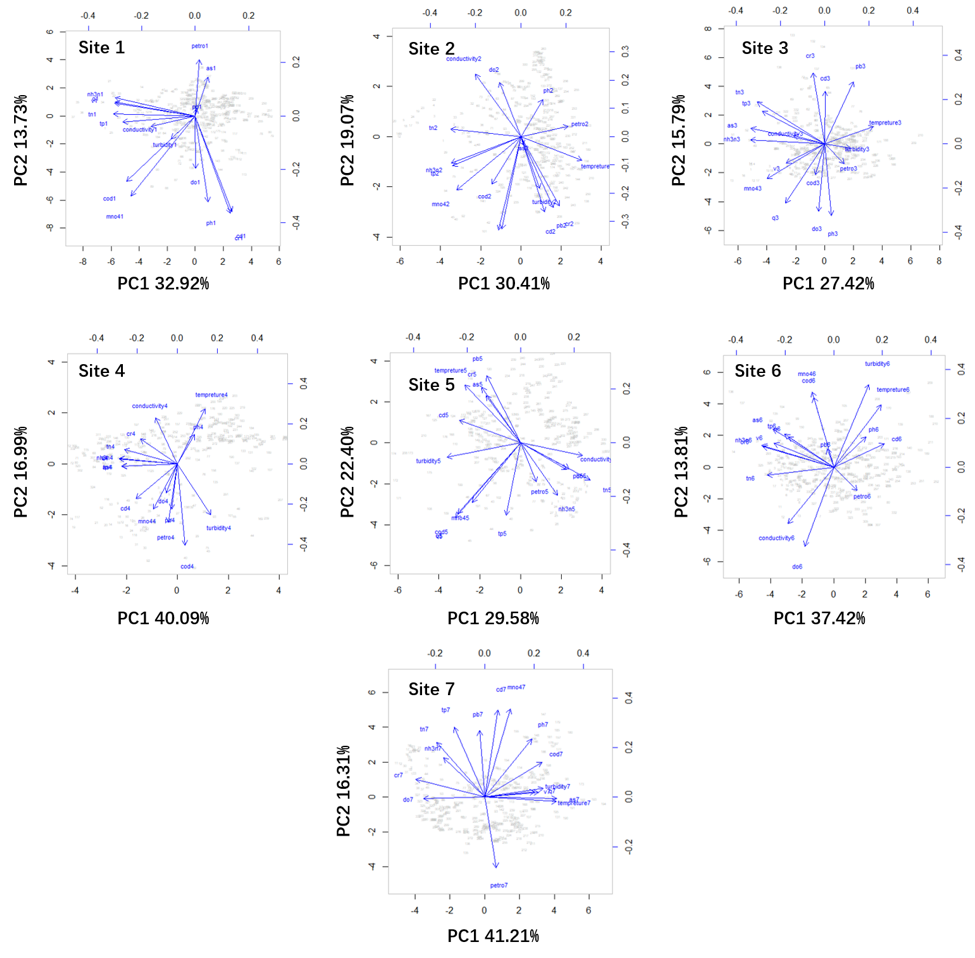

Supplement: Supplementary file 1 — Supplementary Information 1. [file 41598_2021_87671_MOESM1_ESM.docx]
